# Dysregulations of metabolites and gut microbes and their associations in rats with noise induced hearing loss

**DOI:** 10.3389/fmicb.2023.1229407

**Published:** 2023-08-04

**Authors:** Ningning Li, Xiuzhi Zhang, Yanan Cui, Hui Wu, Yue Yu, Shanfa Yu

**Affiliations:** ^1^Department of Pathology, Henan Medical College, Zhengzhou, Henan, China; ^2^Department of Occupational and Environmental Health, College of Public Health, Zhengzhou University, Zhengzhou, Henan, China; ^3^Henan Institute for Occupational Health, Zhengzhou, Henan, China; ^4^National Institute for Occupational Health and Poison Control, Chinese Center for Disease Control and Prevention, Beijing, China; ^5^School of Public Health, Henan Medical College, Zhengzhou, Henan, China

**Keywords:** noise, microbiome, metabolome, hearing loss, correlation analysis

## Abstract

**Background:**

Noise exposure could lead to hearing loss and disorders of various organs. Recent studies have reported the close relations of environmental noise exposure to the metabolomics dysregulations and gut microbiota disturbance in the exposers. However, the associations between gut microbial homeostasis and the body metabolism during noise-induced hearing loss (NIHL) were unclear. To get a full understanding of their synergy in noise-associated diseases, it is essential to uncover their impacts and associations under exposure conditions.

**Methods:**

With ten male rats with background noise exposure (≤ 40 dB) as controls (Ctr group), 20 age- and weight-matched male rats were exposed to 95 dB Sound pressure level (SPL) (LN group, *n* = 10) or 105 dB SPL noise (HN group, *n* = 10) for 30 days with 4 h/d. The auditory brainstem response (ABR) of the rats and their serum biochemical parameters were detected to investigate their hearing status and the potential effects of noise exposure on other organs. Metabolomics (UPLC/Q-TOF-MS) and microbiome (16S rDNA gene sequencing) analyses were performed on samples from the rats. Multivariate analyses and functional enrichments were applied to identify the dysregulated metabolites and gut microbes as well as their associated pathways. Pearson correlation analysis was performed to investigate the associations of the dysregulations of microbiota and the metabolites.

**Results:**

NIHL rat models were constructed. Many biochemical parameters were altered by noise exposure. The gut microbiota constitution and serum metabolic profiles of the noise-exposed rats were also dysregulated. Through metabolomics analysis, 34 and 36 differential metabolites as well as their associated pathways were identified in LN and HN groups, respectively. Comparing with the control rats, six and 14 florae were shown to be significantly dysregulated in the LN group and HN group, respectively. Further association analysis showed significant correlations between differential metabolites and differential microbiota.

**Conclusion:**

There were cochlea injuries and abnormalities of biochemical parameters in the rats with NIHL. Noise exposure could also disrupt the metabolic profiles and the homeostatic balance of gut microbes of the host as well as their correlations. The dysregulated metabolites and microbiota might provide new clues for prevention of noise-related disorders.

## Introduction

Besides its negative effects on auditory system of the exposers, noise exposure, especially long-term exposure, has also been reported to be harmful to other organs and systems. Environmental noise and its associated stress could disrupt the metabolism of the exposers (Schmidt, 2020; Oh and Yoo, 2022; Zhang Y. et al., 2022) and lead to the cardiovascular diseases (Fu et al., 2022; Lucas et al., 2022; Sivakumaran et al., 2022; Zhou et al., 2022), nervous and immune system disorders (Quigley and Lewis, 2022; Onishchenko et al., 2023), as well as dysfunctions of digestion (Liang et al., 2021; Zhang Y. et al., 2022). Recently, noise exposure was found to be associated with the imbalance of the host gut microbiota (Berlow et al., 2021; Chi et al., 2021). Chronic noise exposure was found to have promotive roles in the development of Alzheimer’s disease (AD) and diabetes through the dysregulation of host gut microbiota (Verhaar et al., 2021; Di et al., 2022; Park and Wu, 2022; Wang et al., 2022). To provide new clues for the prevention and treatment of noise related disorders, it is necessary to investigate the dysregulations of metabolism and host gut microbes and explore their associations.

In recent metabolomic studies, intestinal microorganisms were shown to perform their functions through the secretion of metabolites. Metabolites in the blood of the host can in turn interact with the intestinal flora through the intestinal-liver circulation and even the blood–brain barrier to regulate the health of the host (Delzenne et al., 2019; Parker et al., 2020). In a study of arsenic exposed mice, imbalances in the gut flora were reported to have effects on metabolome regulations (Lu et al., 2014). The correlation of the gut microbiota with the metabolome was also confirmed in a clinical study on colorectal cancer (Yang et al., 2019). Microbes participated in the pathogenesis of disease through complex interactions with the host metabolism and immune regulation. Although it has been shown that noise exposure alters the host metabolic profile and causes dysbiosis of the gut flora, comprehensive explanation for the correlation between imbalances in gut microbial homeostasis and metabolic disorders in rats during noise-induced hearing loss (NIHL) development is lacking.

In this study, we applied metabolomics and microbiome approaches to explore the effects of chronic noise exposure on metabolites in host serum as well as on gut microbes. The correlations of the dysregulated metabolites with the dysregulated flora were also investigated to uncover their potential interactions. We hope the results would provide new clues for noise-associated study, new indicators of noise-associated dysfunctions, as well as new potential therapeutic targets for noise-associated diseases.

## Materials and methods

### Animals and noise exposure

30 male SPF Wistar rats (body weight: 160-180 g; age，4–5 weeks old) were purchased from Beijing Vital River Laboratory Animal Technology Co., Ltd. (Beijing, China). The animal license was NO. SCXK (jing) 2016–0006. The rats were housed with controlled light (12 h light/dark cycle), constant temperature (20–26°C), and ambient humidity (40–70%) before being used for experiments (Experimental Animal Center, Tianjin Institute of Health and Environmental Medicine, China). Based on the current upper limit of the safety allowance recommended by the U.S. Occupational Safety and Health Administration (OSHA),^1^ we chose a noise level of 95 dB sound pressure level (SPL) for 4 h per day (4 h/d). To investigate the impact differences between noise exposure of different intensities, 105 dB SPL for 4 h/d was also used for comparison. As a rat was found to have middle ear infection before the experiment, it was excluded and only 29 rats were included in this study. After 1 week of acclimatization, the rats were numbered according to their body weight and the ones with similar weight were then randomly divided into three groups: Ctr group (exposed to background noise ≤40 dB SPL, n = 9), LN group (exposed to 95 dB SPL white noise, *n* = 10) and HN group (exposed to 105 dB SPL white noise, *n* = 10). As reported by Arifin and Zahiruddin (2017), minimum sample sizes for ‘group comparison, one-way-analysis of variance’ design were five animals per group to keep degrees of freedom within the range of 10 to 20. Therefore, we met the sample size required for the experiment with nine or ten rats per group. For the rats in LN group and HN group, exposure was for 4 h/d at 8:30–12:30 for 30 days, referred to a previous study (Cui et al., 2012). In Brief, white noise was generated by a generator (BK 3560C; Brüel & Kjær Instruments, Nærum, Denmark). The main spectrum of the noise signal from the generator was in the range of 20–20,000 Hz, amplified by a power amplifier (Yong-Sheng Audio P-150D; The Third Institute of China Electronic Technology Group, Beijing, China), and emitted from a loudspeaker (ZM-16S; Tianjin Zenmay Electroacoustic Equipment Co., Tianjin, China). To exclude confounding factors, food and water in the Ctr group were also the same as the LN and HN groups at the same time.

The experimental proposals and procedures for the care and treatment of the rats were carried out according to the ARRIVE guidelines (Percie du Sert et al., 2020), and were approved by the Institutional Animal Use and Care Committee, Tianjin Institute of Health and Environmental Medicine (Authorization No. IACUC of AMMS-04-2020-063).

### Auditory brainstem response (ABR)

ABR thresholds of rats before and after noise exposure were measured with auditory evoked potential and otoacoustic emission instrument Smart-EP ASSR (Smart-EP Manual Version 3.97 test system, Smart Listening, United States). All the animals were mildly anesthetized (1% Pentobarbital sodium, 70 mg/kg) and placed in the anechoic room. According to the instructions and previous studies (Paciello et al., 2021, 2023), click stimuli and tone bursts of pure tones from 2 k-32 k HZ (Filter bandwidth: 100 ~ 16,000 HZ, stimulation frequency: 21.10/s, scanning time: 12.8 ms, superimposed 1,024 times) were used and the ABR thresholds of the ears were measured. For the detection, high frequency headphones were used to produce the 16 k and 32 k HZ sounds while the ER3 earphone was used to give the 2 k, 4 k and 8 k HZ sounds. The stimulation starts at 90 dB SPL and decreases at 10 dB SPL. When a threshold occurs, the measurement is repeated twice at the threshold of ±5 dB. The ABR thresholds of the rats among different groups were compared with Kruskal-Wallis test and *p* < 0.05 was considered significant.

### Sample collection and processing

After the last noise exposure, the rats were fasted for 12 h. The blood was collected from the abdominal aorta of anesthetized rats (1% pentobarbital sodium, 0.7 mg/10 g body weight) and centrifuged at 3000 rpm for 10 min at low temperature. The serum was collected, quenched in liquid nitrogen, and stored at −80°C for serum biochemical detection and metabolomic analysis. To avoid contamination, fresh feces (5 g) were collected from the rectal end of the rats and stored in frozen storage tubes after liquid nitrogen quenching for 16S rDNA gene sequencing.

The deep anesthetized rats above were then sacrificed by cervical dislocation. The skull was immediately dissected and the cochlea was obtained. The cochlea tissues were pre-cooled with 4% paraformaldehyde and 2.5% glutaraldehyde solution, and then fixed overnight at 4°C. With the labyrinth parts exposed, the specimens were put into 10% ethylenediaminetetraacetic acid (EDTA) for decalcification. When the cochleae were translucent, they were stored in fixative solution at 4°C for subsequent detection.

### Morphology analysis of the cochlea

The decalcified cochlear tissues were subjected to gradient dehydration, transparency, wax immersion, embedding, serial sectioning, and Hematoxylin–Eosin (HE) staining as described in previous studies (Zhao et al., 2021; Li et al., 2023). With light microscopic observation, the morphological features of the cochlear tissues in different groups were visualized and compared. Laser confocal microscopy (Leica TCS SP8, Leica Microsystems, Germany) was also used to observe the morphological changes of the cochleae.

### Biochemical detection

To investigate the effects of noise exposure on liver function, and regulations of glucose and lipids, the serum levels of alanine transaminase (ALT), aspartate transaminase (AST), alkaline phosphatase (ALP), γ-glutamine transferase (γ-GT), total gallbladder juice acids (TBIL), direct bile acids (DBIL), glucose (GLU), glycosylated serum protein (GSP), triglycerides (TG), total cholesterol (CHO), high-density lipoprotein (HDL), low-density lipoprotein (LDL), creatinine (CREA), UREA and uric acid (UA) were detected with full-automatic biochemical analyzer (Catalyst One® Chemistry Analyzer, IDEXX Laboratories Inc., United States) according to the instructions. Kruskal-Wallis test was used for comparisons among different groups and *p* < 0.05 was considered significant.

### Metabolomics analysis

Untargeted metabolomics tests were performed. Due to clotting during sample acquisition, four samples were excluded and eight samples from Ctr group, seven from LN group and eight from HN group were included for analysis. Each 100 μL serum sample was mixed with 400 μL of pre-cooled methanol/acetonitrile (1: 1, v/v) and then centrifuged for 15 min with 14,000 rpm at 4°C. The supernatant was dried in a vacuum centrifuge and hydrop interaction liquid chromatography (HILIC) separation was performed using a 2.1 mm × 100 mm ACQUIY UPLC BEH 1.7 μm column (waters, Ireland). Detailed processes of extraction, detection, and quality control measures for metabolites in UPLC/Q-TOF-MS analysis are described in previous studies (Liu et al., 2020; Zhang X. et al., 2022).

Raw MS data were subjected to peak extraction to obtain information on characteristic peaks such as m/z values. Metabolites were identified by comparison with the database. Finally, the data files were subjected to univariate statistical analysis (Student’s *t*-test and fold change analysis) and multivariate statistical analysis-principal component analysis (PCA) and orthogonal partial least squares discrimination analysis (OPLS-DA) using the “ropls” package in R3.6.1. The variable importance in the projection (VIP) value of each variable in the prediction was calculated by the OPLS-DA model. Significantly different metabolites were identified with the thresholds of VIP > 1, *p* < 0.05 and Fold change (FC) > 1.5 or FC < 0.67.

### Gut microbiome analysis

16S rDNA gene sequencing on rat feces were performed and the differential flora were screened. As some samples were insufficient or failed DNA extraction, only seven samples from Ctr group, six samples from LN group and nine samples from HN group were included for sequencing analysis. DNA was extracted from fecal samples according to the instructions in the fecal DNA extraction kit (E.Z.N.A. ®Stool DNA Kit, D4015, Omega, Inc., United States). Polymerase chain reaction (PCR) products were confirmed by 2% agarose gel electrophoresis and PCR products. DNA was purified with AMPure XT beads (Beckman Coulter Genomics, Danvers, MA, USA) and quantified using Qubit (Invitrogen, USA). PCR amplification of the V3-V4 region of 16S rDNA from fecal samples was performed using primers 341F (5’-CCTACGGGNGGCWGCAG-3′) and 805R (5’-GACTACHVGGGTATCTAATCC-3′). Equal amounts of amplicons were mixed for sequencing. The size and number of amplicon libraries were evaluated on Agilent 2100 Bioanalyzer (Agilent, United States) and Illumina library quantification kit (Kapa Biosciences, Woburn, MA, United States). Libraries were sequenced on the NovaSeq PE250 platform.

Feature tables and feature sequences were obtained by filtering the raw data according to fqtrim (v0.94). Alpha diversity and beta diversity were randomly normalized to the same sequence. The feature abundance was then normalized according to the SILVA (release: 132) classifier. Species diversity and complexity of the samples were analyzed. The alpha diversity indexes including Chao1 index and Shannon index were evaluated with “vegan” R package. Beta Diversity were calculated with the “QIIME2” R package (v3.5.2). Sequence comparisons were performed using the blast method. Feature sequences were annotated with each representative sequence using the SILVA database. Finally, genera with linear discriminant analysis (LDA) values >3 were identified as significantly differential through linear discriminant analysis effect size (LEfSe) analysis. The Kyoto Encyclopedia of Genes and Genomes (KEGG) pathways (level3) associated with the gut microbiota abundances in different groups were predicated with PICRUSt.^2^ The scores of the pathways among the three groups were compared with Kruskal-Wallis test and *p* < 0.05 was considered significant.

### Microbiome-metabolome correlation analysis

To ensure a one-to-one correspondence between metabolites and gut microbes, we analyzed only the omics data of the rats underwent both metabolomic and microbiomic assays for the combined analyses. Only the significant differential bacterial microbiota (LDA value >3 and *p* < 0.05) and metabolites (VIP > 1, *p* < 0.05, and FC < 0.67 or FC > 1.5) were included. Spearman correlation analysis was performed in R and *p* < 0.05 was considered significant. Clustering correlations were performed using the OmicStudio tools at https://www.omicstudio.cn.

## Results

### The ABR dysregulations in noise-exposed rats

**Figure 1:**
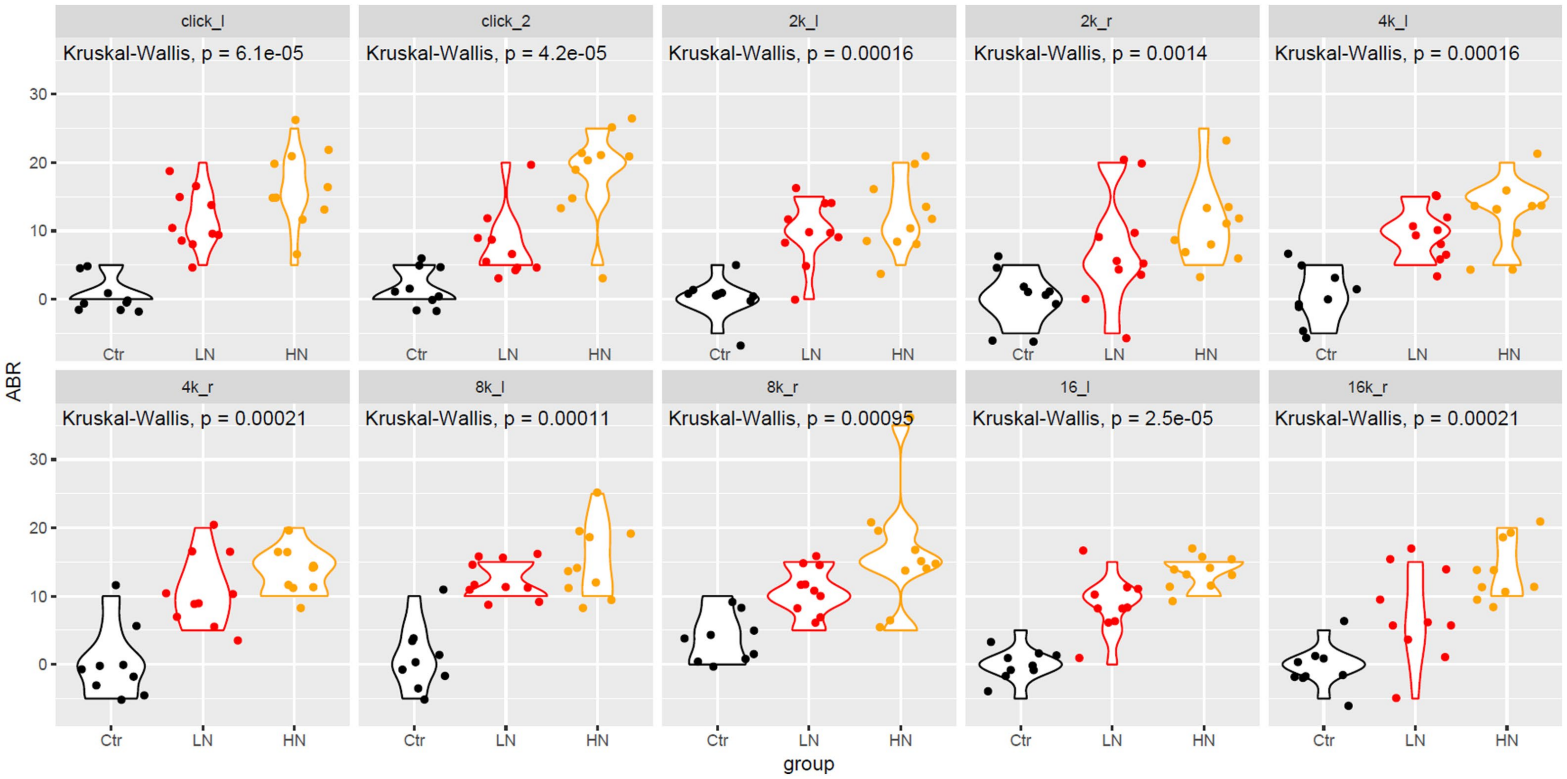
The ABR change differences between different groups after noise-exposure. The x-axis and y-axis indicated the rat groups and the ABR changes, respectively. 2 k, 4 k, 8 k, 16 k represented the frequency of the sound (unit: HZ). The _l and _r indicated the left and right ear, respectively. ABR, auditory brainstem response; Ctr, control; LN, low noise; HN, high noise. Kruskal-Wallis test was used for comparisons. *p* < 0.05 was considered as significant and 0.05 < *p* < 0.1 was considered a trend of significance.

As shown in Figure 1, at all the frequencies, the ABR thresholds of the left and right ears of the Ctr rats presented to be the lowest among the three groups, indicating the significant increase of ABR thresholds of the noise-exposed rats after noise exposure. In contrast, HN rats were shown to have the highest level of ABR thresholds among the three groups. There were increase trends of ABR threshold displacements at click, 4 k, 8 k, and 16 k HZ were also indicated in Ctr, LN, and HN rats. These results indicated the NIHL of the noise-exposed rats and the stronger impacts of higher-intensity noise.

### Microscopic histopathological effects of noise exposure on rat cochleae

**Figure 2:**
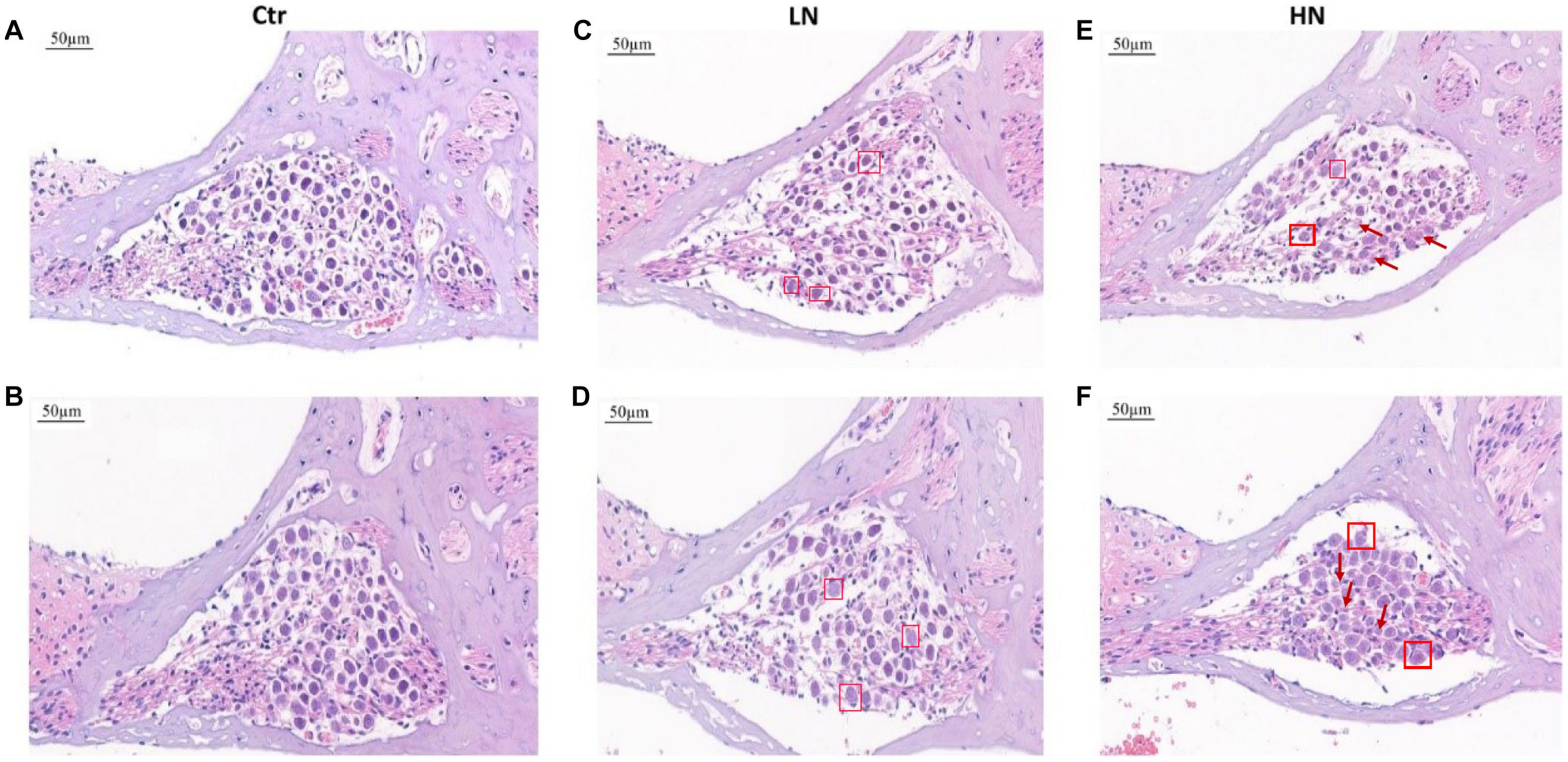
HE staining of cochlear tissues in different groups after noise exposure. **(A,B)** HE staining of cochlear tissues in the Ctr group. **(C,D)** HE staining of cochlear tissues in the LN group. **(E,F)** HE staining of cochlear tissues in the HN group. The swelling nuclei were marked with a square, and loose structures and karyolysis were indicated with arrows. Ctr, control; LN, low noise; HN, high noise.

There were obvious cochlea injuries in the noise-exposed rats. Through HE-staining (Figure 2), it was presented that, comparing with the rats in Ctr group (Figures 2A,B), the spiral ganglion cells in the LN group (Figures 2C,D) decreased with the nucleus swelled and enlarged, and the ratio of cytoplasm and nucleus imbalanced. For the HN group (Figures 2E,F), the spiral ganglion cells presented loose structures, a significant decrease of the nerve cell number, and a disorganized nucleoplasm ratio. Furthermore, in the HN group, a large number of swollen nuclei were also shown and the cell boundaries were obscure. For a few cells, their nucleoli and nuclear membranes also disappeared. These injuries might account for the NIHL in the noise-exposed rats and indicated the stronger impacts of noise-exposure of higher intensity.

**Figure 3:**
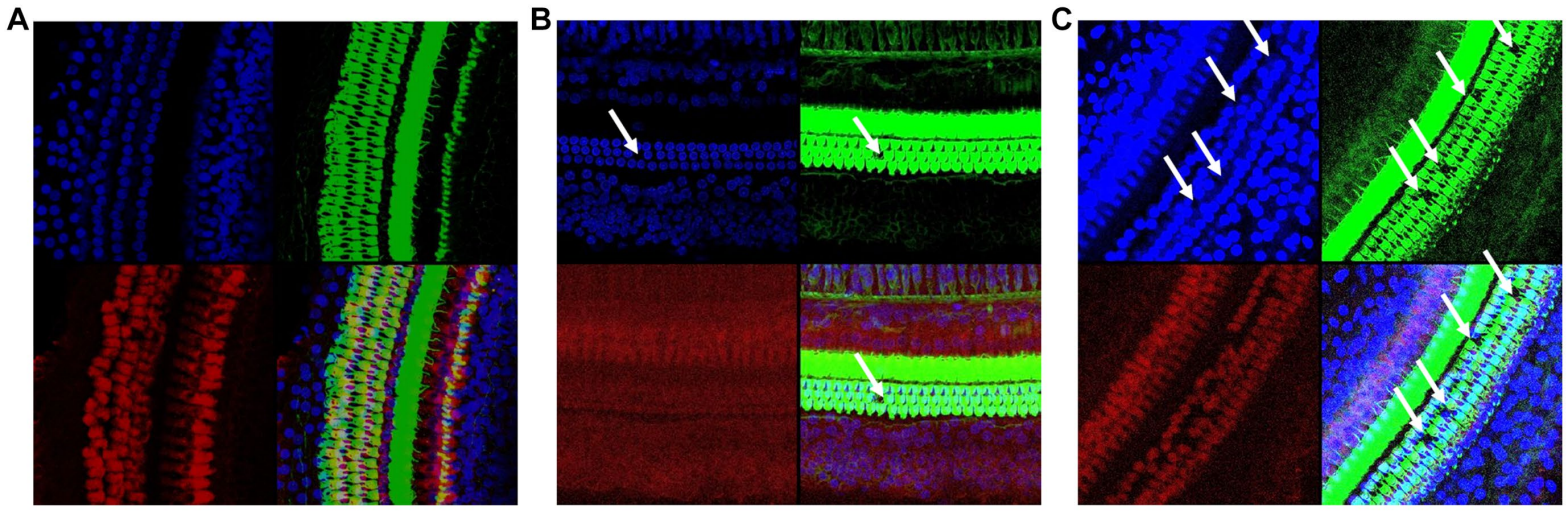
Confocal immunofluorescence of the rat cochlear tissues in different groups. **(A)** Confocal immunofluorescence of the Ctr rat cochlear tissues; **(B)** Confocal immunofluorescence of the LN rat cochlear tissues; **(C)** Confocal immunofluorescence of the HN rat cochlear tissues. The cell plasma and nuclear parts were labeled with green and blue, respectively.

Confocal laser microscopy observation presented similar results. As shown in Figure 3. In the Ctr group (Figure 3A), the cells were arranged neatly. In contrast, external hair cell deletion and nuclear solidification were shown in the LN group (Figure 3B). For the HN group (Figure 3C), the arrangement of the external hair cells was disordered and most of cells presented nuclear displacement, nuclear solidification, nuclear fragmentation, and nuclear lysis, indicating the death and deletion of the external hair cells. However, no significant deletions and misalignment of internal auditory hair cells were shown in the three groups.

### Serum biochemical dysregulations in noise-exposed rats

**Figure 4:**
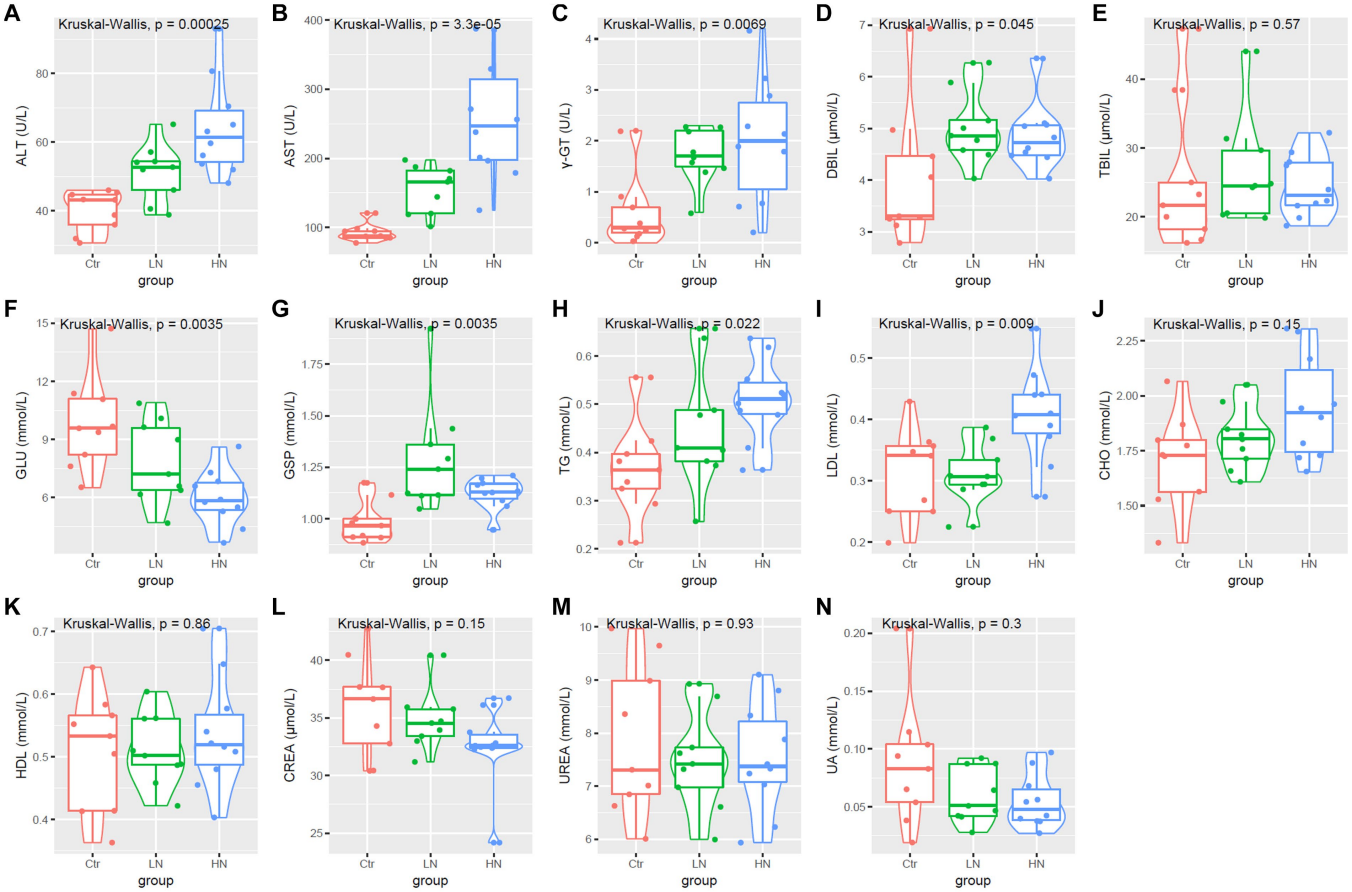
Serum biochemical parameters **(A–N)** comparisons among different groups. Ctr, control; LN, low noise; HN, high noise. Kruskal-Wallis test was used and *p* < 0.05 was considered significant.

As shown in Figures 4A–C, the levels of ALT, AST, and γ-GT were higher in the LN and HN groups that in the control group. Furthermore, the HN presented highest ALT and AST levels among the three groups. Although no significant TBIL was shown among the three groups (Figure 4E), the DBIL were also shown to be higher in the noise-exposed LN and HN rats than the controls (Figure 4D). These results indicated the potential liver injuries of the rats due to noise-exposure, especially in the HN group. The levels of serum glucose and glycosylated serum protein (GSP) also appeared to be dysregulated in the noise-exposed rats (Figures 4F,G), indicating the impact of noise exposure on glucose metabolism. The levels of blood lipids were also affected by the noise-exposure. As shown in Figures 4H,I, TG and LDL increase were shown in noise-exposed rats. Considering the associations of LDL and TG with atherosclerosis (AS) (Yan et al., 2022; Yu et al., 2022), it was indicated that higher noise might be a risk factor of AS. As no significant difference of CHO and HDL was shown among the three groups (Figures 4J,K), the selectiveness of the effects of noise exposure on blood lipids was indicated. Interestingly, no significant difference of CREA, UREA and UA were shown between different groups (Figures 4L–N).

### Effects of noise exposure on serum metabolites

To understand the changes in the metabolic profiles of rat serum after noise exposure, we performed a metabolomic analysis. Pearson correlation analysis on quality control (QC) samples presented a correlation coefficient of *R* > 0.9 in the negative (Supplementary Figure S1A) and the positive (Supplementary Figure S1B) mode, indicating good reproducibility of the experiments. The metabolites and their levels in different groups were shown in Supplementary Tables S1, S2.

**Figure 5:**
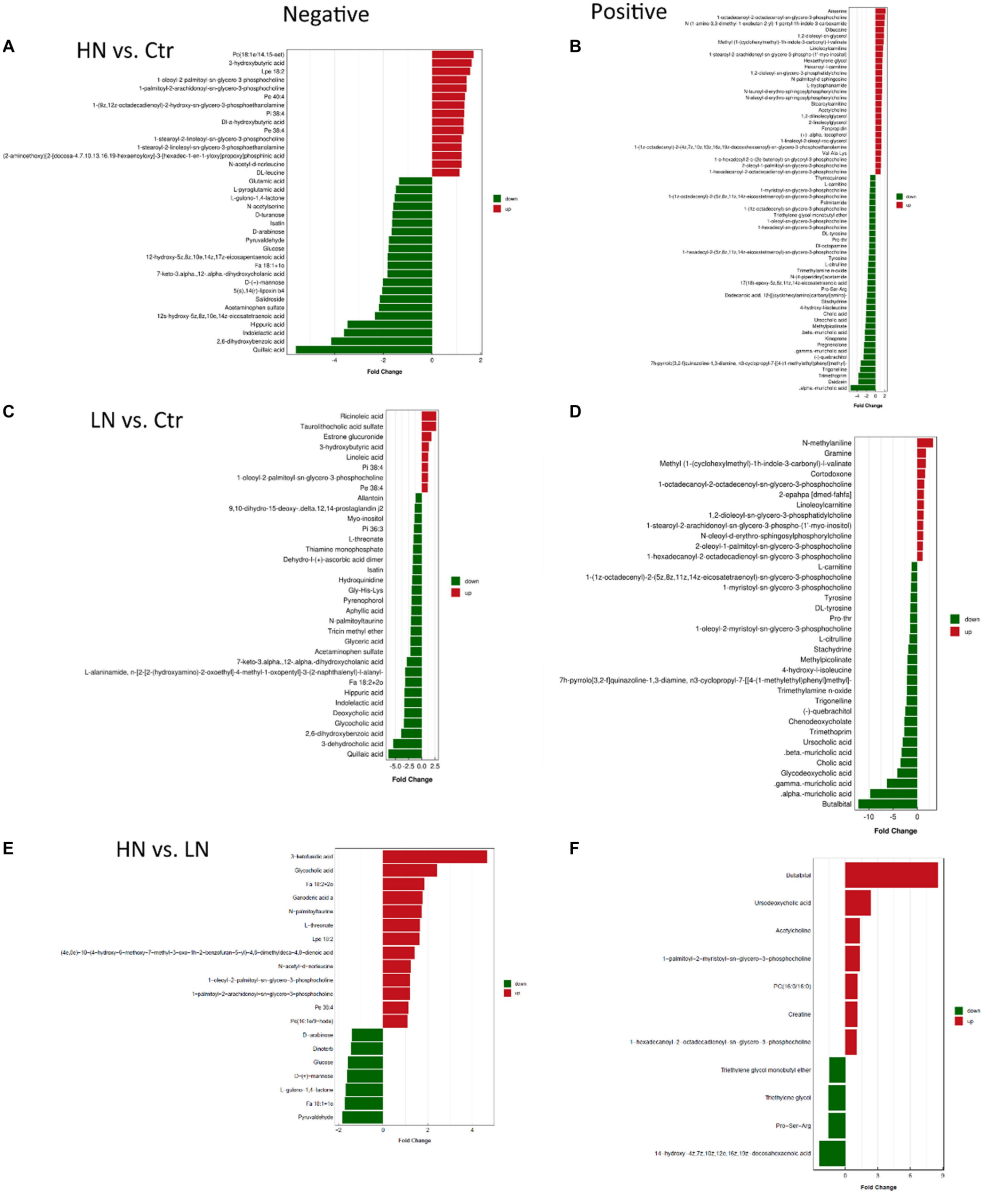
Differential metabolites between different groups. **(A,B)** The differential metabolites between HN and Ctr in the negative and positive mode, respectively. **(C,D)** The differential metabolites between LN and Ctr in the negative and positive mode, respectively. **(E,F)** The differential metabolites between HN and LN in the negative and positive mode, respectively. HN, high noise; LN, low noise; Ctr, control.

Through OPLS-DA (Supplementary Figure S2), each two of the three groups could be discriminated clearly, indicating the significant differences of metabolism between the three groups. Through Wilcoxon test, the metabolic differences between each two of the three groups were investigated. With the threshold of VIP >1 and *p* < 0.05, the differential metabolites were identified (Figure 5). There were 36 (up-regulated: 15, down-regulated: 21) (Figure 5A) and 62 (up-regulated: 27, down-regulated: 35) (Figure 5B) differential metabolites between HN and Ctr groups in the negative and positive modes, respectively. Comparing with the control samples, 34 metabolites (up-regulated: 8, down-regulated: 26) (Figure 5C) and 36 metabolites (up-regulated: 12, down-regulated: 24) (Figure 5D) were found to be dysregulated in LN samples in the negative mode and positive mode, respectively. There were common dysregulated metabolites in the LN and HN groups. In fact, four metabolites (1-oleoyl-2-palmitoyl-sn-glycero-3-phosphocholine, 3-hydroxybutyric acid, Pi 38:4 and Pe 38:4) were upregulated while seven metabolites (Indolelactic acid, Hippuric acid, 2,6-dihydroxybenzoic acid, 7-keto-3.alpha.,12-.alpha.-dihydroxycholanic acid, Acetaminophen sulfate, Isatin, and Quillaic acid) were shown to be downregulated in the two noise-exposed groups. Furthermore, comparing with LN samples, 20 metabolites (negative mode: 13, positive mode: 7) were higher while 11 metabolites (negative mode: 7, positive mode: 4) were lower in HN samples (Figures 5E,F). The common dysregulated metabolites were also provided in the Supplementary Tables (Fig 5_information_supple).

**Figure 6:**
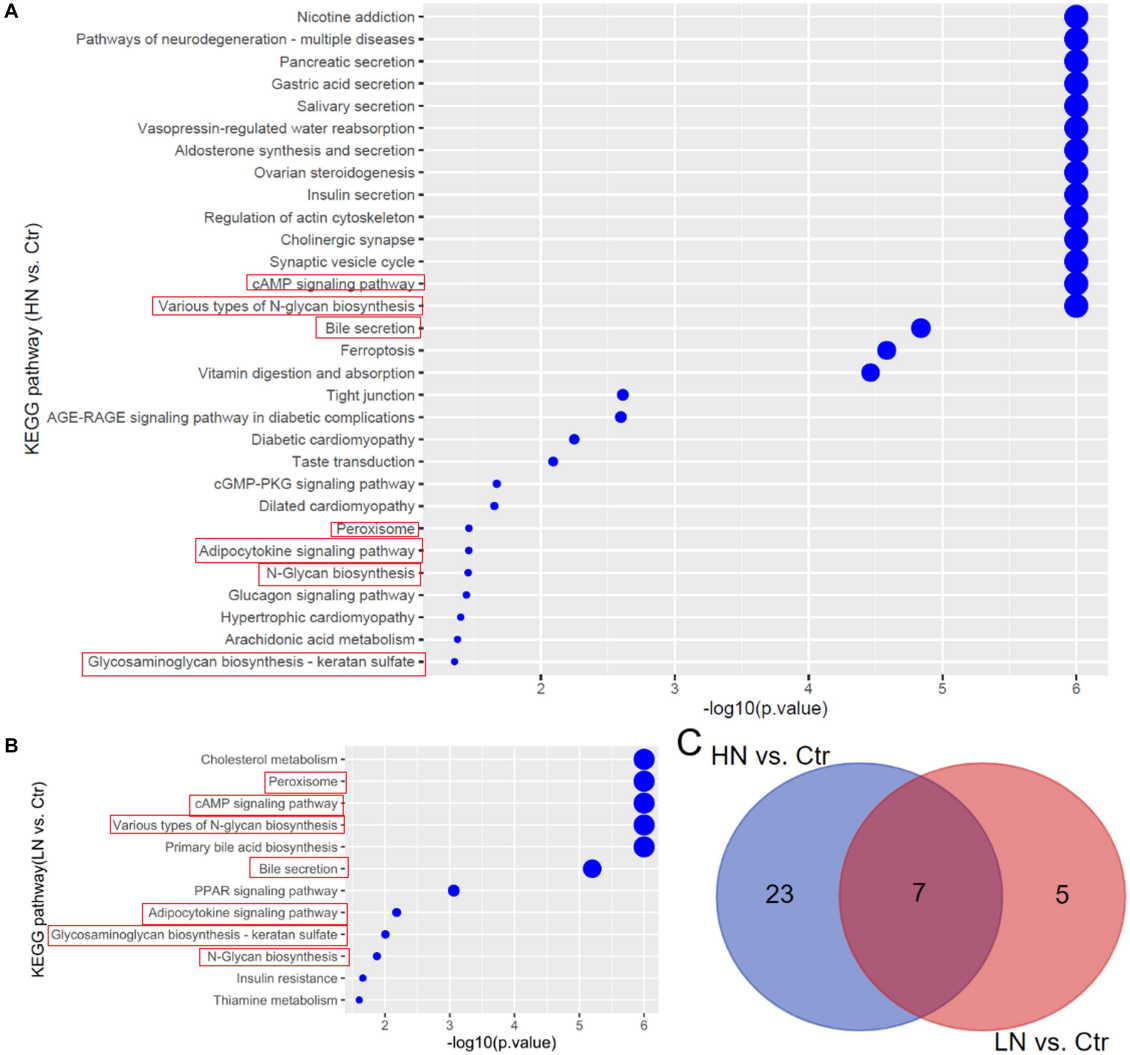
KEGG pathway enrichments of the dysregulated metabolites in noise-exposed rat groups. **(A)** Dysregulated metabolites-associated pathways in HN group. **(B)** Dysregulated metabolites-associated pathways in LN group. **(C)** The common pathways in HN and LN groups due to noise exposure. Ctr, control; LN, low noise; HN, high noise.

Through KEGG pathway enrichment analyses, the dysregulated metabolites in HN and LN groups were shown to be associated with 30 and 12 biological processes, respectively (Figures 6A,B). Noticeably, there were seven pathways (Glycosaminoglycan biosynthesis - keratan sulfate, N-Glycan biosynthesis, Adipocytokine signaling pathway, Peroxisome, Bile secretion, Various types of N-glycan biosynthesis, and cAMP signaling pathway) consistently dysregulated due to noise-exposure in the HN and LN groups while the other processes were different (Figure 6C).

### Effects of noise exposure on intestinal microbial communities

The 16 s rDNA sequencing data was deposited in the NCBI Sequence Read Archive (SRA, http://www.ncbi.nlm.nih.gov/sra), with the accession number PRJNA967148. Through 16S rDNA gene sequencing analyses, a total of 14,358 operational taxonomic units (OTUs) were identified. As shown in Supplementary Figure S3, there were 1,423 common OTUs between Ctr and LN groups, 1,470 common OTUs between Ctr and HN groups, 1,494 common OTUs between LN and HN groups, and 1,032 common OTUs in the three groups. In contrast, the differences among the three groups were also shown and there were 4,025, 3,239, and 4,731 specific OTUs for Ctr group, HN group, and LN group, respectively. The group-specific OTUs indicated the impact of noise exposure of different intensity on the gut microbiome of the hosts.

**Figure 7:**
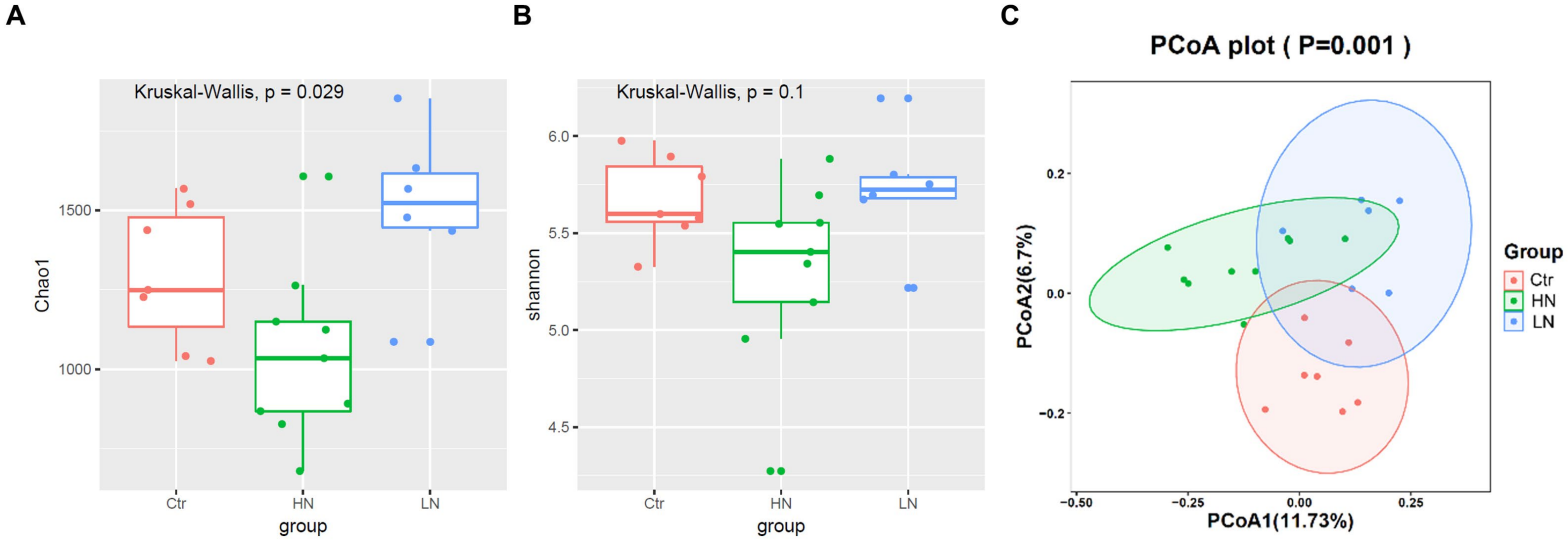
α and ß diversity comparisons of gut microbiota between different groups. **(A,B)** Chao1 index and Shannon index comparisons between different groups. **(C)** ß diversity comparisons among noise-exposed groups (HN and LN) and the control group. Ctr, control; LN, low noise; HN, high noise. Wilcoxon test and PCA analysis were used and *p* < 0.05 was considered significant.

Through Kruskal-Wallis test, the Chao1 index presented significant difference among the three groups (Figure 7A), indicating that different noise exposure with different levels might have different effects on the host gut microbiota. In Figure 7B, the Shannon index of HN group was shown to have a lower trend to be the lowest among the three groups, indicating the decrease trend of microbiota diversity (abundance and richness) in HN group. In Figure 7C, PCA analysis was shown to be able be discriminate the three groups, indicating the significant difference of the ß diversity in the three groups.

**Figure 8:**
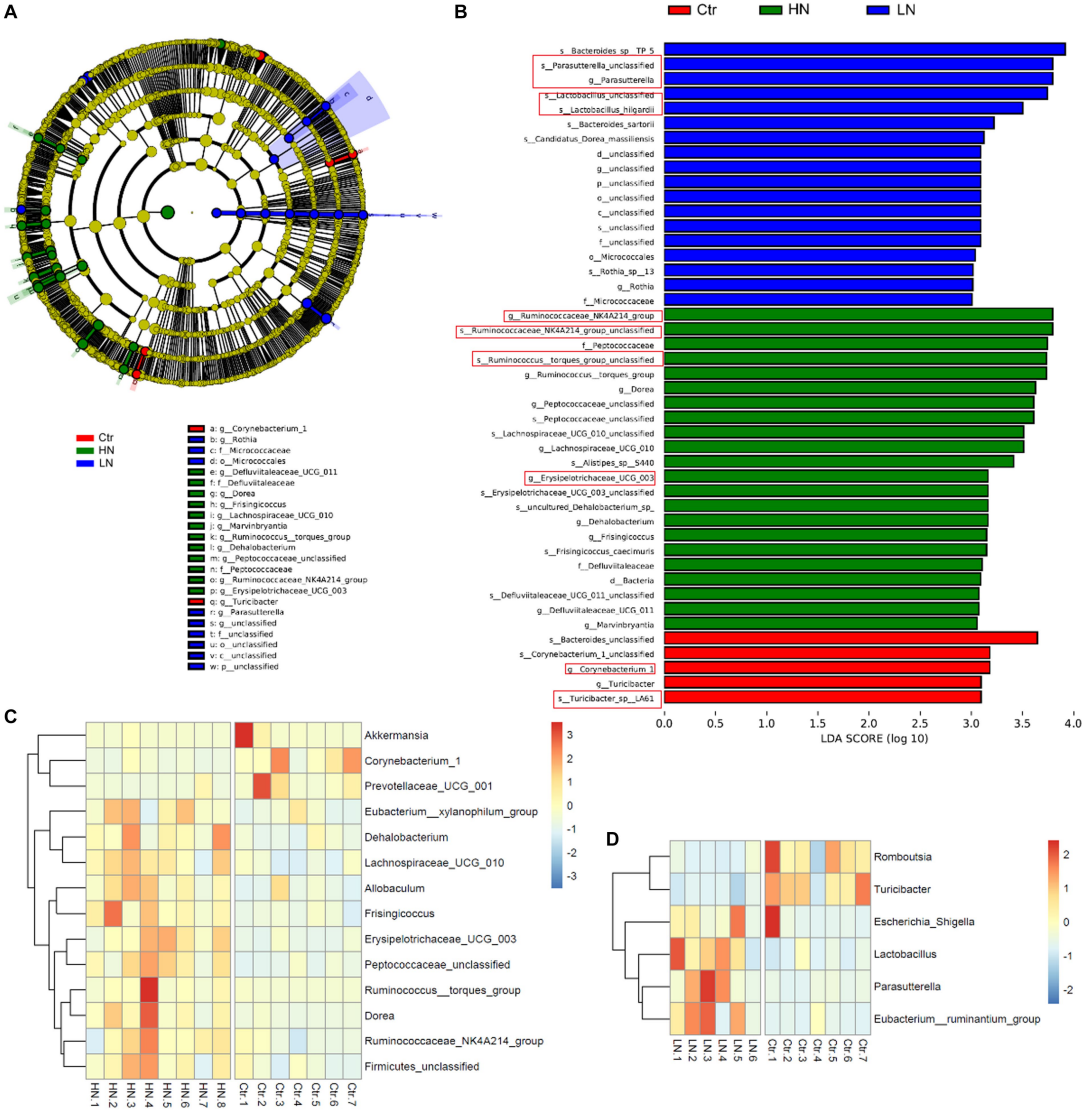
LDA Effect Size (LEfSe) analysis of the gut microbiota in different groups. **(A)** The cladogram of the gut microbiota in the samples. **(B)** The significant biomarkers with LDA score ≥ 3 in different groups. **(C)** The profiles of significantly dysregulated genera (LDA value>3 and *p* < 0.05) in HN group than Ctr group. **(D)** The profiles of significantly dysregulated genera (LDA value>3 and *p* < 0.05) in LN group than Ctr group. Ctr, control; LN, low noise; HN, high noise. The red squares indicated the key microbat for each group. LEfSe analysis and Wilcoxon test were used for analysis. LDA value>3 and *p* < 0.05 were considered statistically significant.

Through LEfSe analysis, the microbiotas with significant abundance differences among different groups were identified. As shown in Figure 8A, the red-, green-, and blue-labeled microbial species were shown to be enriched in the control group, HN group, and LN group, respectively. The potential biomarkers with LDA values >3 was also shown Figures 8B. These results indicated the complex impacts of noise exposure on gut microbiota in the host. Comparing with the Ctr group, there were 14 and six significant microbial genera with LDA value >3 and *p* < 0.05 in HN group (Figure 8C) and LN group (Figure 8D), respectively. It was indicated that noise exposure of different intensity might lead to different impacts on gut microbiotas of the host.

**Figure 9:**
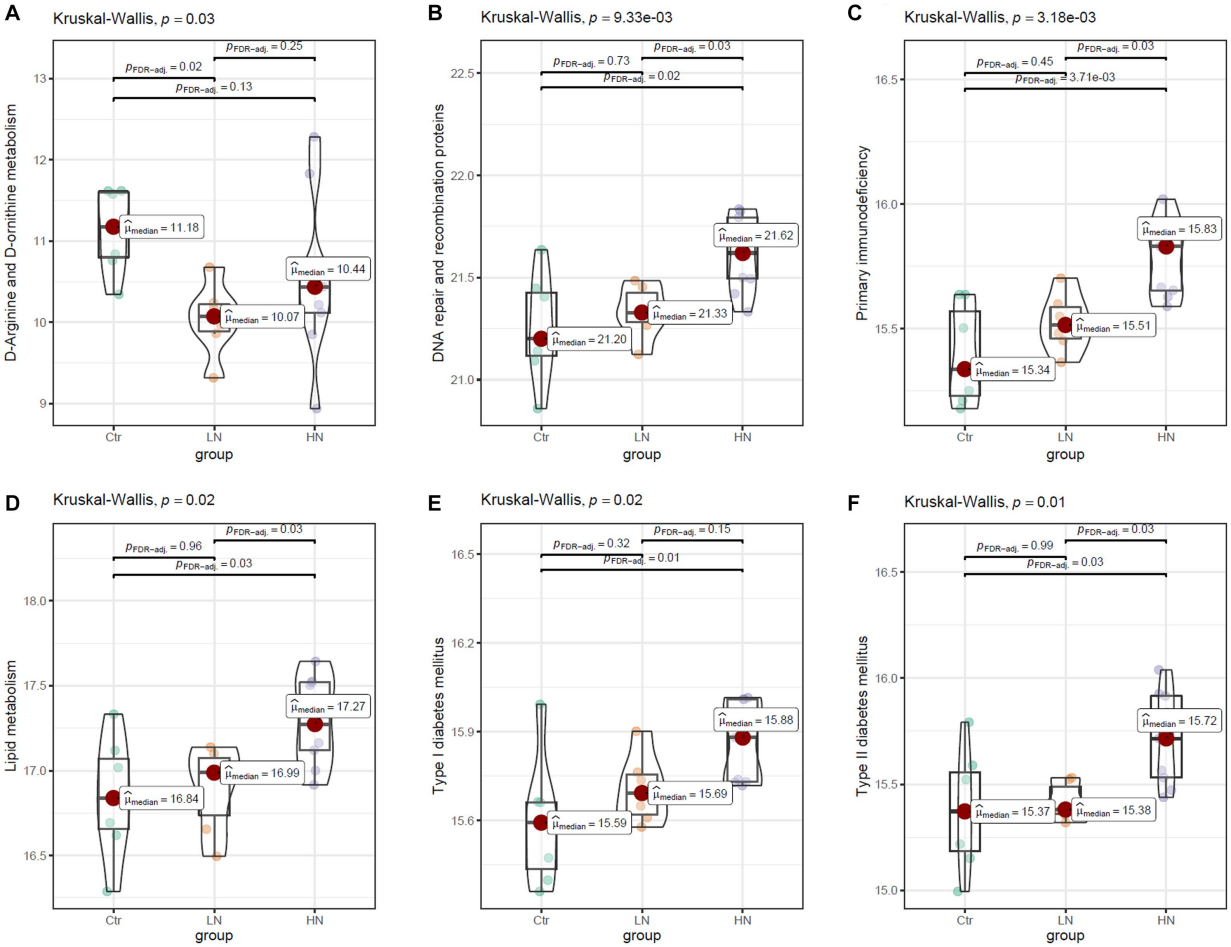
The comparisons of KEGG pathway between different groups. **(A,B)** The higher levels of mineral absorption and D-arginine and D-ornithine metabolism in the LN group than the Ctr group. **(C,D)** The higher levels of primary immunodeficiency and lipid metabolism in the HN group than the Ctr group and the LN group. **(E,F)** The higher levels/trend of type I and type II diabetes mellitus in HN group than the Ctr group and LN group. Ctr, control; LN, low noise; HN, high noise. Kruskal-Wallis test was used for comparisons. *p* < 0.05 was considered significant and 0.05 < *p* < 0.1 was considered a significant trend.

The scores 283 KEGG pathways were predicated (Supplementary Table S3) and compared between the three groups. There were 49.1% (139/283) of the pathways were shown to be dysregulated after noise exposure (Supplementary Table S4). As shown in Figure 9A, the scores of D-arginine and D-ornithine metabolism were shown to be lowest in LN rats among the three groups. For many pathways, highest scores were shown in the HN rats. The representative pathways were shown in Figures 9B–F. Figure 9B presented highest scores of DNA repair and recombination proteins in HN rats, indicating the increase of DNA repair activity due to noise exposure. The highest scores of primary immunodeficiency in HN group (Figure 9C) indicated the potential damage by high-intensity noise exposure to the immunity of exposers. The highest activity of lipid metabolism (Figure 9D) was consistently with the increased TG level in the HN group. The highest scores of type I (Figure 9E) and type II (Figure 9F) diabetes mellitus was consistent the dysregulated serum of GLU and GSP levels in the noise-exposed rats above, implicating the impacts of noise exposure on the GLU regulation.

### Potential relevance of microbiota to metabolites

**Figure 10:**
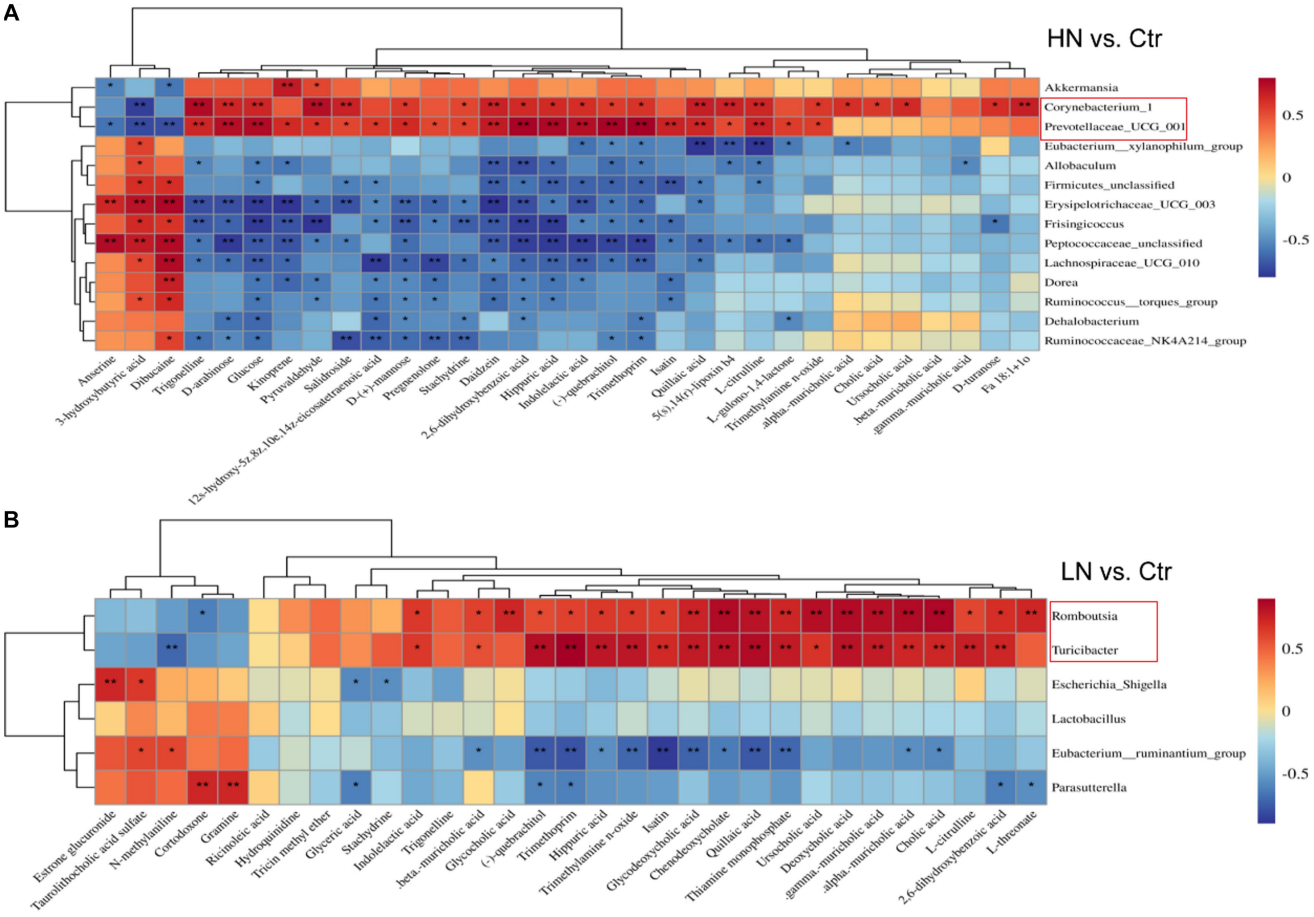
Pearson correlations of dysregulated metabolites and the significant gut microbiota in the noise-exposed rats. **(A)** Correlation of dysregulated metabolites and different microflora in HN group. **(B)** Correlations of dysregulated metabolites and significant microflora in LN group. The depth of color in the figure represented the strength of correlation. ^*^, *p* < 0.05, ^**^, *p* < 0.01.

The associations between the relative abundance of significantly dysregulated genera above and the expression of significantly dysregulated metabolites were analyzed. In the HN group (Figure 10A), *Corynebacterium_1* and *Prevotellaceae_UCG_001* were shown to be significantly correlated with 23 and 25 metabolites, respectively. *Erysipelotrichaceae_UCG_003, Frisingicoccus*, and *Peptococcaceae_unclassified* were significantly correlated with 20, 14, and 21 metabolites, respectively. In the LN group (Figure 10B), *Romboutsia* and *Turicibacter* were significantly associated with 21 (20 positive and 1 negative correlation), and 19 (18 positive and 1 negative correlation) metabolites, respectively. While *Eubacterium_ruminantium_group* was negatively correlated with 12 metabolites and two positively correlated. These results suggest close relations between the gut microbiota and the metabolic profiles of the rats during noise exposure.

## Discussion

Long-term noise exposure could disrupt the metabolic profiles of the exposers, leading to the development of a variety of diseases (Miao et al., 2021). In our previous study, noise exposure was shown to be associated with the dysregulations of multiple biological processes in the noise-exposed steel workers with and without NIHL (Zhang X. et al., 2022). Here, we successfully constructed NIHL rat models and the dysregulated pathways associated with noise exposure of different intensities were uncovered. Consistently, similar pathways were shown several biosynthesis-related and digestion-absorption associated processes were also included. In the present study, we also found the dysregulations of microbiome in the rats with NIHL. In recent years, noise-induced imbalances in host gut flora were proposed to be a trigger for immune and metabolic disorders (Cui et al., 2016, 2018). The metabolic dysregulation and immune dysfunctions were also shown in the noise-exposed rats. Furthermore, we combined the metabolomic and the microbiome analyses and presented the significant correlations of the dysregulated metabolites and gut flora imbalance due to noise-exposure. These results might provide new clues for prevention of noise-associated disorders and further study of noise exposure.

The associations between noise exposure and NIHL has been reported in plenty of studies. As there might be noise exposure of various intensities in different occupational environments, it was necessary to investigate the common and specific effects of noise exposure of different intensities. Here, we presented that both the low and high noise exposure could lead to NIHL, and the higher noise exposure could bring more severe injuries of the cochlea, more significant dysregulations of the biochemical parameters. These results indicated the variety of impacts of noise exposure and importance of avoiding it and reducing its intensity. Through metabolomic analyses, we identified the differential metabolites between in the noise-exposed rats and their associated processes. It was reported that bile acids (BAs) were important in body metabolism and closely related to lipid metabolism, glucose metabolism and immune response of the body (Sipka and Bruckner, 2014; Shapiro et al., 2018; Li et al., 2021). Here, both in the LN and HN groups, Bas were shown to be dysregulated. Their dysregulations in the LN and HN rats might be important for NIHL occurrence and dysfunctions of many other noise-associated disorders. Besides the common dysregulated metabolites, there were also LN- and HN-specific metabolic dysregulations. Furthermore, in contrast to the common pathways in the metabolomic analysis and the common dysregulations of the biochemical parameters, the KEGG pathways drawn from microbiome analysis were significant different between LN and HN groups. It was speculated that the impacts of noise-exposure of different intensities on the gut microbiotas was more specific. The similarities and differences in these results further suggested the need for multi-omics and multi-level analyses to fully understand the effects of noise exposure.

Although the links of noise-induced flora imbalance with a variety of metabolic diseases have been reported, the interpretations of microbiome and metabolome alterations under chronic noise exposure were unclear. In order to get a comprehensive understanding of intestinal microorganisms and metabolites altered by noise exposure, we investigated the associations between host metabolism and gut microbes. The significant correlations between the dysregulated metabolites and genera under different noise intensities were uncovered. *Parasutterella*, which was closely associated with intestinal inflammation, has recently been found to have a potential role in bile acid maintenance and cholesterol metabolism [18]. Here, *Parasutterella* was found to be correlated with many metabolites in LN rats. Its increase in LN rats might be associated with the dysregulations of DBIL and LDL in the group. It was reported that reduced abundance of *Romboutsia* and *Turicibacter* also plays an important role in diabetes-induced cognitive decline (AlShawaqfeh et al., 2017; Gao et al., 2019). In this study, we also shown the decrease of the two genera in LN rats, which might be associated with the glucose dysregulation in the group. It was reported that significant alterations in the abundance of *Prevotellaceae_UCG-001* and *Corynebacterium_1* at the genus level were indicators of possible abnormalities in immune inflammation and sugar and lipid metabolism (Xiao et al., 2020). Here, interestingly, the positive correlations of *Romboutsia* and *Turicibacter* with BAs in the LN rats indicated their coordination in the processes of metabolism, consistent with the results in Theriot C M. study (Theriot et al., 2016). We also found the decrease of *Prevotellaceae_UCG-001* and *Corynebacterium_1* in the HN group. Their significant correlations with many metabolites were presented, which might account for the dysregulations of immunity and glucose metabolism in the HN rats.

It was reported that the gut flora could regulate the size of the BAs pool and the ratio of each BA component, and modify the BA molecule (Vítek and Haluzík, 2016). In turn, BAs could also regulate the homeostasis of the intestinal flora, preventing bacterial translocation and enhancing mucosal barrier defenses (Funabashi et al., 2020). Disturbances in circadian rhythms and worsening environmental factors were also found to be able to alter bile acid metabolism, alter intestinal flora and even trigger liver and intestinal inflammation (Meng et al., 2019). All of these results indicated the bidirectional interactions between bile acid synthesis and gut microbiotas. Considering the significant correlations between dysregulated metabolism and gut microbiotas in the noise-exposed rats and their associated pathways, we speculated that during NIHL and other noise-related disorders, there might be intimate interactions between metabolism and gut microbiota. The dysregulated metabolites and microbiotas and their associations might provide new clues for noise-exposure study. There were also limitations in our study. As only male rats were included, the impacts of sex differences on the metabolome and gut microbiome could not be evaluated. And, further study was needed to uncover the specific interactions between the dysregulated metabolites and the gut genera under noise exposure of different intensities.

## Conclusion

In summary, we successfully constructed NIHL rat models with noise-exposure of low and high intensities. The high-intensity noise exposure presented more serious injuries on the cochleae, more obvious ABR changes, and more significant biochemical parameter dysregulations than the low-intensity noise exposure. Long-term noise exposure could alter the serum metabolic profiles and the gut microbiota of the Wistar rats. There were significant correlations between differential flora and differential metabolites during noise exposure. The high-intensity noise exposure might lead to dysregulations of more metabolites and gut microbiotas than the low-intensity ones. The results in this study provided possible directions for the prevention and control of noise pollution. The dysregulated biochemical parameters, metabolites and gut genera might provide new markers for the diagnosis of noise-associated disorders and new therapeutic targets for their treatments.

## Data availability statement

The datasets presented in this study can be found in online repositories. The names of the repository/repositories and accession number(s) can be found in the article/Supplementary material.

## Ethics statement

The animal study was approved by the Institutional Animal Use and Care Committee, Tianjin Institute of Health and Environmental Medicine. The study was conducted in accordance with the local legislation and institutional requirements.

## Author contributions

NL and XZ conceived and designed study, performed research and wrote the paper. YC and YY conceived or designed study, technical and material support. XZ and SY reviewed the intellectual contents of the manuscript. HW provided the technical and material support. SY designed experiments, provided administrative, technical and material support, and obtained the funding. All authors contributed to the article and approved the submitted version.

## Funding

This work was supported by the National Natural Science Foundation of China (No. 81872574).

## Conflict of interest

The authors declare that the research was conducted in the absence of any commercial or financial relationships that could be construed as a potential conflict of interest.

## Publisher’s note

All claims expressed in this article are solely those of the authors and do not necessarily represent those of their affiliated organizations, or those of the publisher, the editors and the reviewers. Any product that may be evaluated in this article, or claim that may be made by its manufacturer, is not guaranteed or endorsed by the publisher.

## Footnotes

^1^http://oshanoise.com/osha_standard.html

^2^http://huttenhower.sph.harvard.edu/galaxy/
