# Supplementary material for: Dysregulations of metabolites and gut microbes and their associations in rats with noise induced hearing loss
Source: Front Microbiol. 2023 Aug 4;14:1229407. doi: 10.3389/fmicb.2023.1229407 (PMC10436299; doi:10.3389/fmicb.2023.1229407)
Supplement: Supplementary file 2 [file Data_Sheet_1.PDF]

**Supplementary figures**

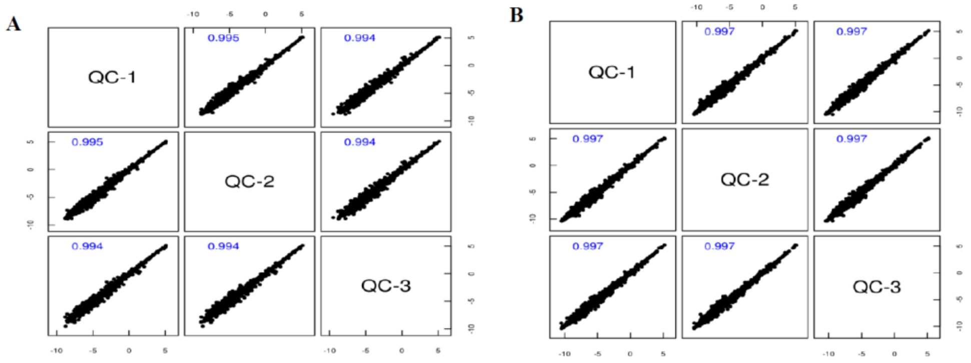


**Figure S1.** Pearson correlation analysis on quality control samples in the negative (A) and positive modes (B). QC, quality control.

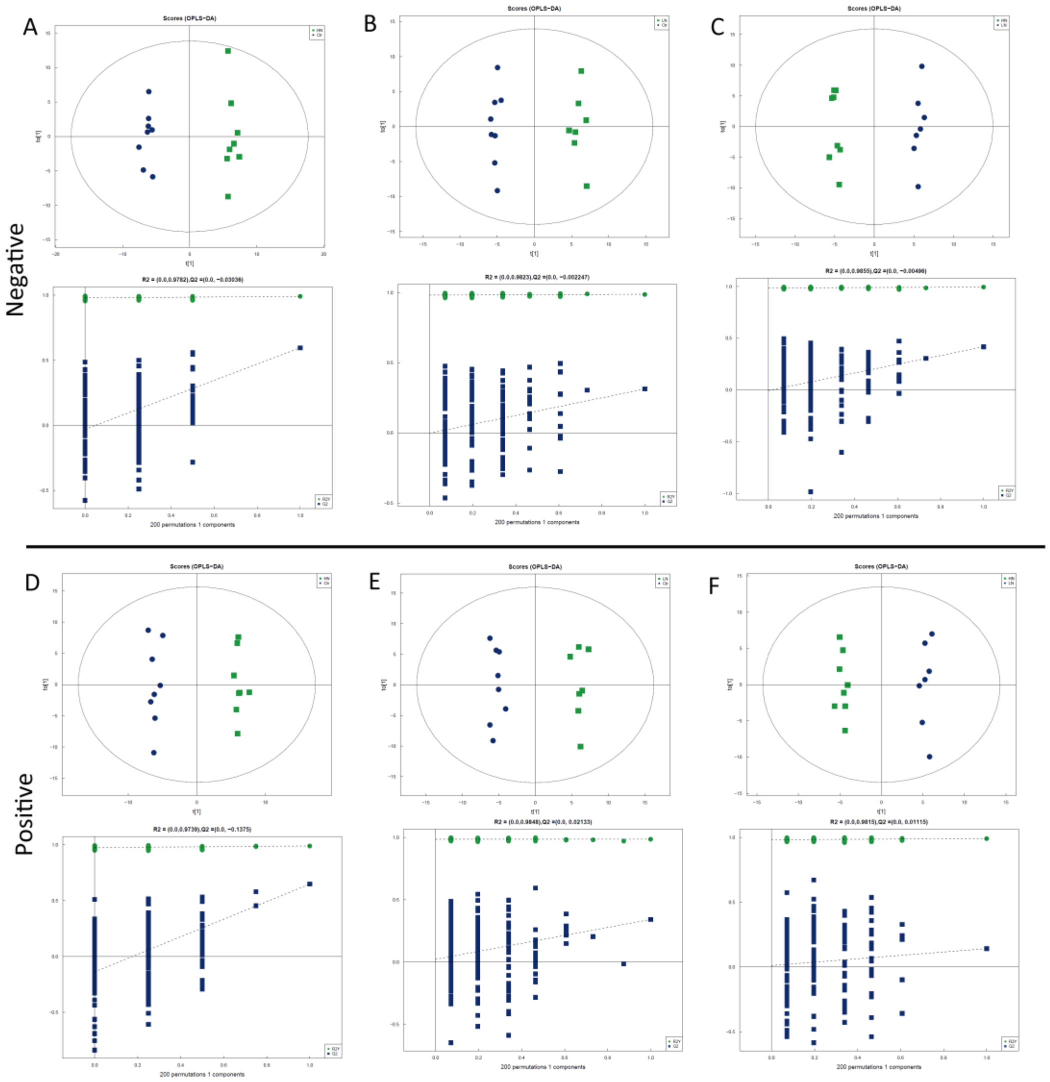


**Figure S2.** OPLS-DA of the plasma metabolites between HN，LN and control groups. (A-C) OPLS-DA score plots of HN vs. Ctr, LN vs. Ctr, and HN vs. HN in the negative node, respectively. (D-F) OPLS-DA score plots of HN vs. Ctr, LN vs. Ctr, and HN vs. HN in the negative node, respectively.

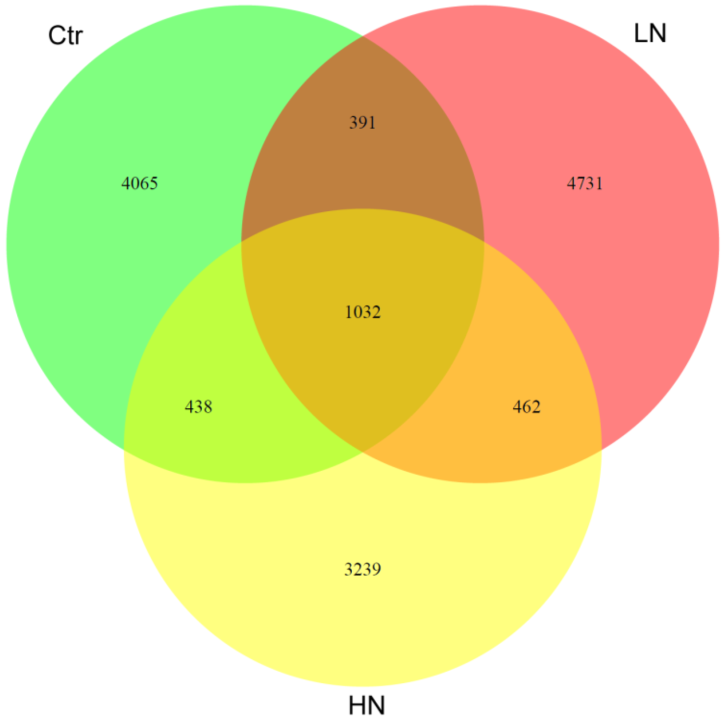


**Figure S3.** The operational taxonomic units (OTUs) identified in different groups. Ctr, control, LN, low noise; HN, high noise.
